# Duplication, divergence and persistence in the Phytochrome photoreceptor gene family of cottons (*Gossypium *spp.)

**DOI:** 10.1186/1471-2229-10-119

**Published:** 2010-06-20

**Authors:** Ibrokhim Y Abdurakhmonov, Zabardast T Buriev, Carla Jo Logan-Young, Abdusattor Abdukarimov, Alan E Pepper

**Affiliations:** 1 Center of Genomic Technologies, Academy of Sciences of Uzbekistan. Yuqori Yuz, Qibray region Tashkent, 111226 Uzbekistan; 2 Department of Biology, Texas A&M University, College Station, Texas 77843, USA

## Abstract

**Background:**

Phytochromes are a family of red/far-red photoreceptors that regulate a number of important developmental traits in cotton (*Gossypium *spp.), including plant architecture, fiber development, and photoperiodic flowering. Little is known about the composition and evolution of the phytochrome gene family in diploid (*G. herbaceum*, *G. raimondii*) or allotetraploid (*G. hirsutum*, *G. barbadense*) cotton species. The objective of this study was to obtain a preliminary inventory and molecular-evolutionary characterization of the phytochrome gene family in cotton.

**Results:**

We used comparative sequence resources to design low-degeneracy PCR primers that amplify genomic sequence tags (GSTs) for members of the *PHYA*, *PHYB/D*, *PHYC *and *PHYE *gene sub-families from A- and D-genome diploid and AD-genome allotetraploid *Gossypium *species. We identified two paralogous *PHYA *genes (designated *PHYA1 *and *PHYA2*) in diploid cottons, the result of a Malvaceae-specific *PHYA *gene duplication that occurred approximately 14 million years ago (MYA), before the divergence of the A- and D-genome ancestors. We identified a single gene copy of *PHYB*, *PHYC*, and *PHYE *in diploid cottons. The allotetraploid genomes have largely retained the complete gene complements inherited from both of the diploid genome ancestors, with at least four *PHYA *genes and two genes encoding *PHYB*, *PHYC *and *PHYE *in the AD-genomes. We did not identify a *PHYD *gene in any cotton genomes examined.

**Conclusions:**

Detailed sequence analysis suggests that phytochrome genes retained after duplication by segmental duplication and allopolyploidy appear to be evolving independently under a birth-and-death-process with strong purifying selection. Our study provides a preliminary phytochrome gene inventory that is necessary and sufficient for further characterization of the biological functions of each of the cotton phytochrome genes, and for the development of 'candidate gene' markers that are potentially useful for cotton improvement via modern marker-assisted selection strategies.

## Background

Phytochromes are specialized photoreceptors that perceive and interpret light signals from the environment to regulate virtually all aspects of plant development, including seed germination, chloroplast development, tropisms, shade avoidance responses, floral initiation, circadian rhythms, pigmentation, and senescence [1-3]. The phytochromes have a primary role in sensing red (R) and far-red (FR) light, and also play a role in the perception of blue (B) and ultraviolet (UV) light [4]. The active phytochrome molecule consists of a large (~110 kDa) apoprotein bound to a phycobilin chromophore [5,6]. The phytochrome apoproteins are encoded by a small gene family in all plant taxonomic divisions, including parasitic plants, mosses, cryptogams, and green algae [7-13]. In angiosperms, the phytochrome apoprotein genes have been classified into four or five gene sub-families based on sequence similarity to the five phytochrome genes of Arabidopsis: *PHYA*, *PHYB*, *PHYC*, *PHYD*, and *PHYE *[14,15]. All five Arabidopsis phytochromes share an amino acid sequence similarity of 46-56%, with the exception *PHYB *and *PHYD*--which are the result of recent gene duplication and share ~80% amino acid identity [14,16]. Thus, the five Arabidopsis genes are often assigned to four subfamilies: *PHYA*, *PHYB*/*D*, *PHYC*, and *PHYE *[17]. The Arabidopsis *PHYB/D *subfamily is more closely related to *PHYE *gene (~55% nt identity) than to the *PHYA *and *PHYC *genes (~47% nt identity), which together form a separate ancient evolutionary clade [13,14].

Having presumably arisen by gene duplication and subsequent subfunctionalization and/or neofunctionalization, the phytochrome gene family *in toto *performs a complex network of redundant, partially redundant, non-overlapping, and in some cases antagonistic regulatory functions throughout plant development [18-35]. For example, all Arabidopsis phytochromes play diverse and interacting roles in photoperiodic regulation of floral initiation. *PHYA*, *PHYB*, *PHYD *and *PHYE *act partially redundantly in the light-dependent entrainment of the circadian clock [35,36], which in turn regulates transcription of the floral inducer *CONSTANS *(*CO*) in a circadian manner [37]. In Arabidopsis, *PHYA*, in conjunction with blue-light dependent cryptochrome photoreceptors *CRY1 *and *CRY2*, promotes flowering by inhibiting the degradation of *CO *protein, while *PHYB *acts antagonistically to stimulate *CO *degradation [38]. In addition, *PHYB*, *PHYD *and *PHYE *act partially redundantly as repressors of flowering that are dependent on R/FR ratio [19,28,30,39]. In this role, *PHYB *also acts downstream of *CO *as a negative regulator of transcription of the 'florigen' molecule *FT *(the target of *CO*) in a tissue specific manner [40]. Mutant analyses indicate that *PHYC *also plays a role in photoperiodic flowering [31,41]. Further, genetic variation at the *PHYC *locus underlies some of the natural phenotypic variation in flowering time in Arabidopsis [42,43].

In angiosperms, the composition of phytochrome gene family varies significantly among taxonomic lineages. Although a single *PHYA *gene is present in most flowering plants, some plant families, such as carnation (Carryophyllaceae) and legumes (Fabaceae), have two distinct *PHYA *genes [10]. Similarly, several plant lineages have gained multiple *PHYB*-like genes through independent gene duplications of *PHYB *[10,14,16,44-47]. For example, tomato has two *PHYB *genes (designated *PHYB*1 and *PHYB*2) that are not directly orthologous to Arabidopsis *PHYB *and *PHYD*, respectively [44]. While most angiosperms have a single *PHYC *gene, species in some families such as Fabaceae and Salicaceae appear to have lost *PHYC *during evolution [10,47]. Although a single *PHYE*-like gene is present in most flowering plants, *PHYE *is completely absent in poplar (Salicaceae), in the Piperales, and some monocots such as maize [10,47]. Finally, the novel *PHYF *subfamily, which groups with *PHYA*/*C *clade, has been identified in tomato [44].

Little is known about the composition of the phytochrome gene family in cultivated cottons or their wild relatives (*Gossypium *spp.) in the Malvaceae family. This is despite the fact that physiological experiments suggest that phytochromes regulate economically important aspects of cotton development, including drought resistance, seed germination, plant architecture, photoperiodic flowering, and fiber elongation [48-51]. For example, R/FR photon ratio influences the length and diameter of developing seed fiber; fibers exposed to a high R/FR photon ratio during development were longer than those that received lower R/FR ratio, implicating the involvement of a phytochrome [50,51].

While modern domesticated varieties of the major cultivated cottons *G. hirsutum *L. and *G. barbadense *L. exhibit photoperiod independent flowering, wild and 'primitive' accessions of *G. hirsutum *and *G. barbadense *flower under short-day photoperiodic control [52,53]. An understanding of the molecular-genetic basis of differences in photoperiodic flowering in cottons will accelerate strategies for improvement of cultivated varieties through the introgression of valuable genetic traits from wild germplasm [52,53]. In this regard, it is important to note that mutational changes in phytochrome function have been implicated in the loss of photoperiod sensitivity in several major crops including sorghum, barley, rice, and soy [54-57].

A thorough characterization of the phytochrome gene family in cotton species is necessary for understanding the molecular basis of photoperiodic flowering, the influences of light quality on cotton fiber elongation, and other aspects of cotton development. Any inventory of phytochrome genes of cottons is complicated by the fact that the major cultivated species, *G. hirsutum *and *G. barbadense *are allotetraploids. Diploid species in the genus *Gossypium *are categorized into eight genome groups (designated A through G, and K) based on cytogenetic and phylogenetic criteria [58-62]. The old-world A genome group and the new world D genome group diverged from each other on the order of 1-7 MYA [61], then underwent hybridization and polyploidization creating an AD allopolyploid lineage ancestral to *G. hirsutum *(designated AD_1_) and *G. barbadense *(designated AD_2_) on the order of 1 MYA [62,63].

In this study, we utilized a PCR-based approach with low-degeneracy primers to obtain gene fragments, or 'genome sequence tags' (GSTs) that yield an initial description of the composition and evolution of the phytochrome gene family in the New World allotetraploid cottons *Gossypium hirsutum *and *G. barbadense*, and in the Old-World diploids *G herbaceum *L. and *G. raimondii *Ulbr., which are considered to be extant relatives of the A- and D-genome diploid ancestors (respectively) of the allotetraploid lineage. This study provides a necessary foundation for studies of the specific biological functions of each of the phytochrome genes in cotton species, and helps to illuminate the evolutionary patterns of duplicated genes in complex genomes, as well as the evolutionary history of the world's most important fiber crop species.

## Results

Because our results were derived from PCR, our inventory of the phytochrome gene family in *Gossypium *spp. is provisional. All sequences have been submitted to GenBank (accession numbers HM143735-HM143763).

### Phytochrome hinge amplification using 'universal' primers

**Figure 1:**
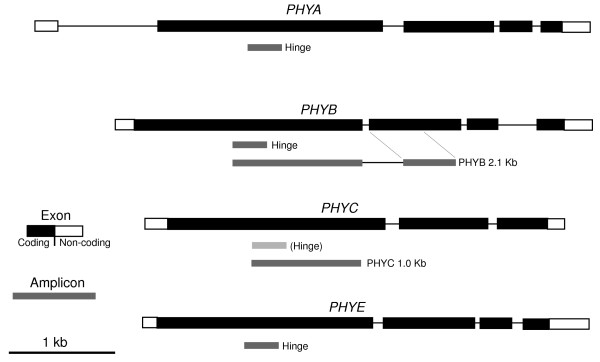
**Gene diagrams and PCR amplicons used in this study**. Model gene structures are derived from *Arabidopsis thaliana *annotations (At1g09570, At2g18790. At5g35840, At4g18130). The *PHYB *2.1 kb sequenced fragment represents a composite of several overlapping amplicons.

Between N-terminal 'photoperception domain' and C-terminal 'signaling domain' of the phytochrome apoprotein is a short 'hinge region' (Figure 1) that shows relatively high sequence variation, and has proven useful for characterization of the phytochrome gene complement in a variety of plant species, and for robust phylogenetic analyses [10]. To amplify the hinge region of all cotton phytochromes, we used an alignment of eudicot phytochrome sequences to design a 768-fold degenerate PCR primer (designated PHYdeg-F) based on the conserved HYPATDIP peptide in the N-terminal domain, and a 16,384-fold degenerate PCR primer (designated PHYdeg-R), based on the conserved PFPLRYAC peptide in the C-terminal domain (Table 1).

**Table 1:** Primers used to amplify cotton phytochrome gene family.

Primer name	Sequence 5' to 3'	Fold-degeneracy
PHYdeg-F	CAYTAYYCIGCIACIGAYATHCC	768

PHYdeg-R	CRCAIGCRTAICKARIGGRWAIGG	16,384

PHYABnondeg-F	GCATTATCCTGCTACTACTGATATT	0

PHYAdeg-R	CAWGCATACCTWAGMGGRAAI	64

PHYBdeg-R	AACAACIAIICCCCAIAGCCTCAT	64

1010-F	GTTYTTGTTTAAGCARAACCG	4

1910-R	GAGTCWCKCAGAATAAGC	4

1910-F	AGCTTATTCTGMGWGACTC	4

2848-R	TAACCCKCTTRTTTGCAGTCA	2

PHYC-1R-DFCI	GGTCCGCCTGATTGAGACTGC	0

I corresponds to inosine. R, Y, M, K, S, W correspond to the IUPAC-IUB ambiguity set.

Amplification across the hinge region using Taq DNA polymerase yielded PCR products from all taxa. We cloned the amplification products from each taxon into an *E. coli vector*, then sequenced ~40 clones for each taxon. For all taxa, a majority (>60%) of clones showed the highest similarity in BLAST searches to Arabidopsis *PHYE *(*E *value ~ 1e^-40^). For each taxon, only a minority of clones showed high-scoring similarity to Arabidopsis *PHYA *or *PHYB*. This apparently skewed distribution of amplification products -- observed across all taxa -- suggested an amplification bias in favor of *PHYE *amplicons. No clones were obtained from any taxon that had high-scoring similarity to Arabidopsis *PHYC *or *PHYD*. No new phytochrome sub-families were observed.

### Amplification of the *PHYA *gene sub-family

Because of possible biased amplification, we designed new less-degenerate hinge-region primer sets for the *PHYA, PHYB/D*, and *PHYC *sub-families (Table 1) using available phytochrome sequences from species in the rosid clade, which includes both cotton and Arabidopsis [64,65].

**Figure 2:**
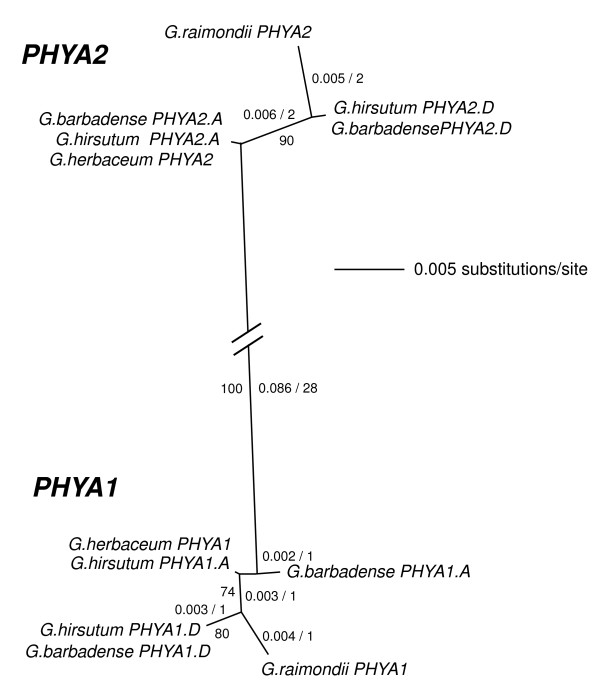
**Unrooted NJ tree of *Gossypium *spp. *PHYA*-related genes based on a ~315 bp consensus alignment of amplification products from the hinge region**. Distances (uncorrected "p") and most parsimonious number of nt changes are indicated for each branch (to the left and to the right of the/, respectively). Branch lengths of less than 0.001 substitutions per site are not shown. Bootstrap support (500 replicates) is indicated where >50%.

The hinge regions of *PHYA *genes were amplified using PHYABnondeg-F and PHYAdeg-R (Table 1), yielding a ~360 bp amplification product from all accessions. In BLAST database searches, all clones had a high-scoring pair relationship with Arabidopsis *PHYA *(*E *value ~ 2e^-63^). Sequences from a total of more than 200 clones across all taxa yielded two distinct consensus contigs from each of the diploids *G. herbaceum *and *G. raimondii*, and four distinct contigs from the allotetraploids *G. barbadense *and *G. hirsutum*. When aligned across all taxa, these contigs yielded a 315 bp consensus alignment that had an average pairwise sequence similarity of 94.6%, with 282 sites (89.5%) identical across all taxa, and no stop codons or indels in any taxa. Distance analysis (Figure 2) showed two well-separated gene sub-clades (100% bootstrap support). These sub-clades were designated *PHYA1 *and *PHYA2*. The level of hinge-region differentiation between these two sub-clades was far greater than that seen in other cotton phytochrome gene sub-families (discussed below), with an uncorrected "p" distance of 0.086, corresponding to 28 nt changes (9%) based on parsimony.

These data indicated that a single *PHYA *gene underwent duplication after the divergence of the cotton and Arabidopsis lineages, but prior to the divergence of A-genome and D-genome lineages, leaving each of the modern diploids in our study (and presumably the ancestors to the AD allotetraploids) with a complement of two *PHYA *paralogs (*PHYA-1 *and *PHYA-2*). Indeed, four distinct contigs were observed in both the inbred *G. hirsutum *cultivar TM-1 and in the doubled-haploid line *G. barbadense *3-79. For each allotetraploid taxon, two contigs fell into each of the *PHYA-1 *and *PHYA-2 *clades (Figure 2). A conservative inventory of available EST sequences indicated that at least two distinct *PHYA *loci are expressed in *G. hirsutum *(Additional file 1).

Within each of the *PHYA1 *and *PHYA2 *clades, the level of nucleotide diversity was very low, with at most four parsimonious nucleotide changes separating each contig. However, within the *PHYA1 *clade, the contigs resolved into two subclades (74% bootstrap support) that each contained a single contig from one of the diploid taxa and one contig from each of the allotetraploids. For example, *G. raimondii *(D-genome) *PHYA1 *grouped in a single contig from each of *G. hirsutum *and *G. barbadense*. Based on this grouping, the latter contigs were assigned the provisional designation of *PHYA1.D*. Similarly, *G. herbaceum *(A-genome) grouped with *G. hirsutum PHYA1.A *and *G. barbadense PHYA1.A*. Based on similar criteria, the *PHYA2 *clade was also divided into *PHYA2.A *and *PHYA2.D *subclades (90% bootstrap support). The phylogenetic resolution of A- and D-genome subclades supported the hypothesis that each of the A- and D-genome diploids contributed both *PHYA1 *and *PHYA2 *to the allotetraploid lineage. Thus, although hinge-region nucleotide diversity within each of the *PHYA1 *and *PHYA2 *clades was low, it was sufficient to resolve a tentative *PHYA *gene complement for each taxon, as well as the pattern of gene inheritance through the allopolyploidization event.

### Amplification of the *PHYB/D *gene sub-family

A ~320 bp fragment from the *PHYB/D *hinge region was obtained by amplification using primers PHYABnondeg-F and PHYBdeg-R (Table 1). Sequences from a total of 80 clones yielded a single consensus contig from each of the diploid cottons *G. herbaceum *and *G. raimondii*, and from the allotetraploid *G. hirsutum*. Two distinct contigs were assembled from clones derived from the allotetraploid *G. barbadense*. These clone sequences shared ~85% nucleotide identity with the Arabidopsis *PHYB *gene and ~78% nt identity with Arabidopsis *PHYD*. All clones had a high-scoring pair relationship with the Arabidopsis *PHYB *gene (*E *value ~ 1e^-71^) as well as significant similarity to the Arabidopsis *PHYD *gene (*E *value ~ 3e^-55^). Consensus sequences were aligned across all taxa, yielding a 319 bp alignment with an average pairwise sequence similarity of 99.8%, with 317 sites (99.4%) identical across all taxa, no stop codons and no indels. Although these data indicated the presence of at least one *PHYB *gene in each of the A- and D-genome diploid plants and in *G. hirsutum*, and at least two genes *PHYB *genes in the *G. barbadense*, the low level of nucleotide differentiation observed within the hinge region yielded insufficient phylogenetic information to characterize the *PHYB *gene complement in any of the study taxa.

To obtain better resolution of the *PHYB *gene complement, additional low degeneracy primers 1010-F, 1910-F, 1910-R, and 2848-R (Table 1) were used along with primer PHYABnondeg-F to create a 2.1 kb long series of overlapping amplicons corresponding to approximately 1.8 kb of the Arabidopsis *PHYB *gene and extending from the hinge, through the first intron and into the second exon (Figure 1). After amplification, cloning and sequencing, the amplicons were assembled for each taxon. In all *Gossypium *taxa examined, the first intron was ~300 bp longer than the first intron of *PHYB *from Arabidopsis.

Unlike the other phytochrome amplicons, we detected a high frequency of putative PCR-mediated recombination events [66] within the *PHYB2.1 *kb fragment from amplifications using *G. barbadense *as template. The recombination detection algorithm RDP3 [67] identified a number of clones resulting from apparent recombination between the A-genome and D-genome derived homeologous sequences, with predicted breakpoints (*P = *0) between nucleotides 1000 and 1700 of the alignment. After omission of these recombinant clones, composite amplicon sequences from each taxon were aligned, creating a consensus alignment of 2,061 bp with 98.8% average pairwise similarity and 2,007 identical sites (97.4%). Overall, the cotton *PHYB *genes shared 65% nucleotide identity with the Arabidopsis *PHYB *ortholog. No stop codons or indels were detected in exon sequences. A 2 bp putative deletion was observed in one contig (designated *PHYB.D*) from *G. hirsutum*. In addition, a 1 bp indel was polymorphic between the PHYB.A and PHYB.D clades. Finally, *PHYB *of *G. raimondii *had an additional 1 bp insertion. All indel polymorphisms were located within first introns.

**Figure 3:**
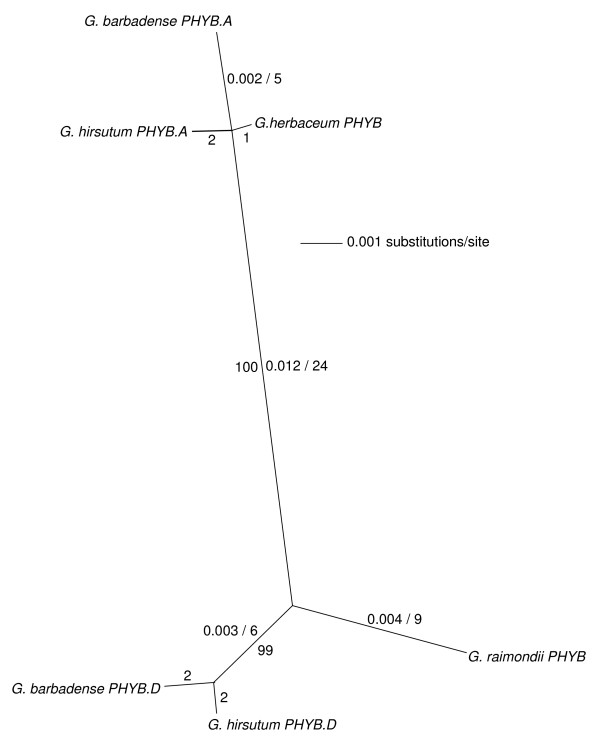
**Unrooted NJ tree of *Gossypium *spp. *PHYB*-related genes based on the consensus alignment of the ~2.1 kb merged amplicons**. Distances (uncorrected "p") and most parsimonious number of nucleotide changes are indicated for each branch (to the left and to the right of the/, respectively). Branch lengths of less than 0.001 substitutions per site are not shown. Bootstrap support (500 replicates) is indicated where >50%.

Detailed phylogenetic analyses of the 2,061 bp contigs from A-, D-, and AD-genome cottons (Figure 3) indicated the presence of least one *PHYB *locus in the two diploid cottons, *G. herbaceum *and *G. raimondii*, and at least two *PHYB *loci in both allotetraploid cottons. The *G. hirsutum *and *G. barbadense *sequence contigs each grouped into two sub-clades (tentatively designated *PHYB.A *and *PHYB.D*). The single *PHYB *contig from *G. herbaceum *was used to define the *PHYB.A *cluster (99% bootstrap support), while the single *PHYB *contig from *G. raimondii *anchored the *PHYB.D *cluster. From these results, we concluded that *PHYB.A *and *PHYB.D*, which shared ~98% nucleotide sequence identity, arose as orthologs at the time of divergence of the A- and D-genome diploid lineages. We surmised that *PHYB.A *was contributed to the allotetraploids via the A-genome ancestor and *PHYB.D *was contributed via the D-genome ancestor. Available EST sequences indicated that at least one *PHYB *locus is expressed in *G. hirsutum *(Additional file 1).

### Amplification from the *PHYC *gene sub-family

Several sets of degenerate primer pairs that were designed on the basis of the conserved HYPATDIP and PFPLRYAC regions -- including several designed from rosid *PHYC *nucleotide sequences -- failed to produce detectable PCR amplification products from the *Gossypium *species tested (data not shown). However, the identification of a small EST clone (GenBank CO121409) with similarity to Arabidopsis *PHYC *(*E *value = 7e^-119^) in a library from *G. raimondii *floral tissue [68], allowed us to design the primer PHYC_1R_DFCI within the C-terminal domain (Table 1). When used in combination with PHYdeg-F, this primer amplified a ~1 kb fragment composed entirely of coding sequence from the first exon of *PHYC*, including the hinge (Figure 1). All clones obtained using this primer pair had a high-scoring similarity to Arabidopsis *PHYC *(*E value *~ 1e^-172^). From these clones, we assembled a single consensus contig from each of the diploid species *G. herbaceum *and *G. raimondii*, and two distinct consensus contigs from each of the allotetraploids *G. hirsutum *and *G. barbadense*. Consensus sequences for each of the putative *PHYC *contigs were aligned across all taxa, yielding a 1,022 bp alignment with an average pairwise sequence similarity of 99.1%, 1,002 sites (98.0%) identical across all taxa, with no indels or stop codons in any taxa.

**Figure 4:**
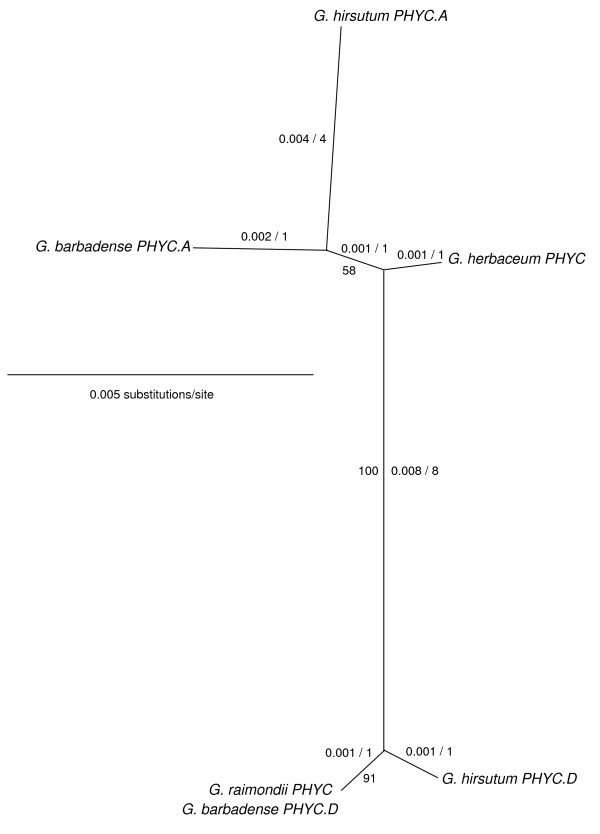
**Unrooted NJ tree of *Gossypium *spp. *PHYC*-related genes based on a 1022 bp consensus alignment of amplification products from primers PHYdeg-F and PHYC_1R_DFCI**. Distances (uncorrected "p") and most parsimonious number of nucleotide changes are indicated for each branch (to the left and to the right of the/, respectively). Bootstrap support (500 replicates) is indicated where >50%.

In phylogenetic analyses (Figure 4), the *PHYC *consensus sequences grouped into two major clades (100% bootstrap support). One of these clades contained the *G. herbaceum *contig and one contig from each of *G. hirsutum *and *G. barbadense*. This clade was designated *PHYC.A*. The other clade, designated *PHYC.D*, included the *G. raimondii *contig along with the other of the two contigs from each of *G. hirsutum *and *G. barbadense*. These data indicated that both the A- and D-genome ancestors had one *PHYC *gene, and that upon hybridization and polyploidization, this gene was contributed from each diploid to the allotetraploid ancestor of *G. hirsutum *and *G. barbadense*.

For comparison with the other phytochromes, we also analyzed a portion of the *PHYC *alignment corresponding to the hinge region only. This alignment was 296 nucleotide pairs in length, with pairwise sequence similarity of 99.0%, 290 sites (98.0%) identical across all taxa, with no indels. Although it encompassed fewer variable nucleotides, NJ analysis of the hinge region alone could be used to differentiate the *PHYC.A *and *PHYC.D *clades (100% bootstrap support) and to infer the composition and evolutionary inheritance of the *PHYC *gene family in cottons (data not shown).

Our failure to obtain *PHYC *hinge amplification with several sets of both universal (e.g. PHYdeg-F/PHYdeg-R) and rosid specific primers was entirely due to substantial nucleotide differentiation in *PHYC*, particularly within the hinge region. For example, the 24 nt long PHYdeg-R primer had six nucleotide mismatches with the cotton *PHYC *genes, including three transitions and three transversions. Five of the six mismatches occurred at what are considered to be invariant (e.g. non-degenerate) nucleotide positions. It should be noted that these divergent nucleotides in the conserved primer-binding site did not alter the amino acid sequence (PFPLRYAC).

### The *PHYE *gene sub-family

**Figure 5:**
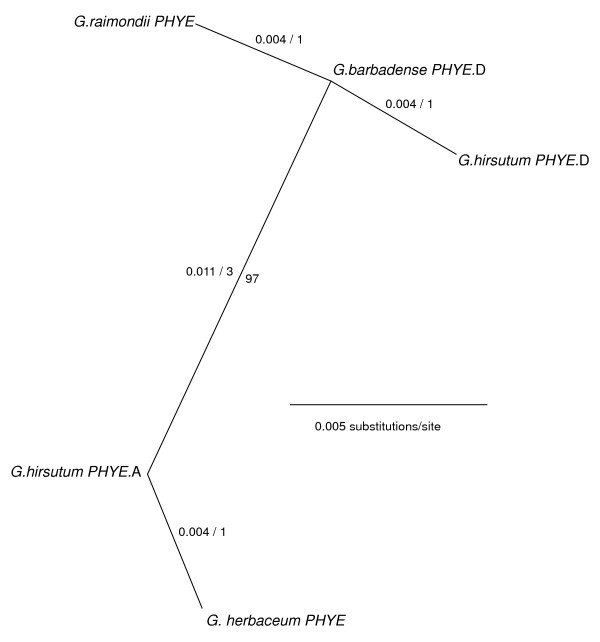
**Unrooted NJ tree of *Gossypium *spp. *PHYE*-related genes based on a 270 bp consensus alignment of amplification products from the hinge region**. Distances (uncorrected "p") and most parsimonious number of nucleotide changes are indicated for each branch. Branch lengths of less than 0.001 substitutions per site are not indicated. Bootstrap support (500 replicates) is indicated where >50%.

*PHYE *hinge region consensus contigs from our study taxa formed a 270 bp alignment with an average pairwise similarity of 98.9%, with 264 (97.8%) invariant sites, no indels, and no stop codons in any taxa. The consensus of the aligned *PHYE *sequences had 80% nucleotide similarity to the corresponding fragment of the Arabidopsis *PHYE *gene. Based on maximum parsimony, nucleotide diversity in the cotton *PHYE *hinge sequences could be explained by a minimum of six nucleotide changes, all of which were synonymous. NJ analysis of the cotton *PHYE *hinge region showed two distinct clades (97% bootstrap support) corresponding to the A- and D-genome derived orthologs (designated *PHYE.A *and *PHYE.D*), a finding consistent with a hypothesis in which each diploid ancestor contributed a single *PHYE *ortholog to the allotetraploid lineage (Figure 5). Interestingly, while two distinct *PHYE *contigs were obtained from *G. hirsutum*, only a single contig, which grouped with the D-genome clade, was obtained from *G. barbadense*. Available EST sequences indicated that at least one *PHYE *locus is expressed in *G. hirsutum *(Additional file 1).

### A global hinge-based alignment of Arabidopsis and cotton phytochromes

**Figure 6:**
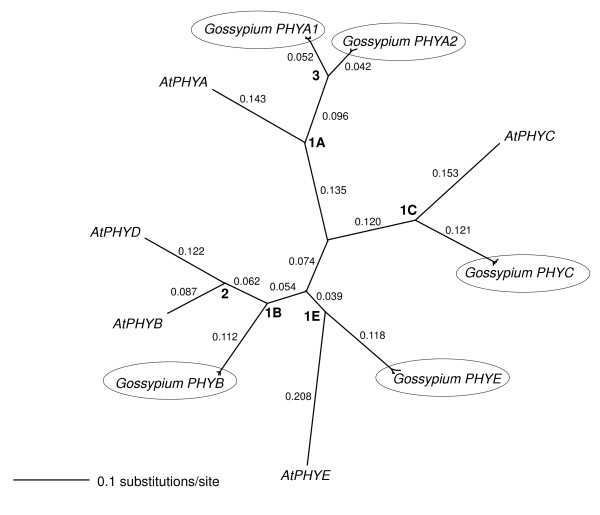
**Unrooted NJ tree of phytochrome genes of *A. thaliana *and cottons (*Gossypium *spp.) based on a 358 bp consensus alignment of amplification products from the hinge regions**. Kimura two-parameter distances are shown for each branch. All internal branches had 100% bootstrap support (500 replicates). 1A, 1B, 1C and 1E denote gene divergence events likely resulting from speciation. 2 and 3 denote gene divergence events likely resulting from gene duplication. All *Gossypium *phytochrome genes are included within clusters (indicated by ovals).

*PHYA*, *PHYB*, *PHYC *and *PHYE *hinge regions from Arabidopsis and *Gossypium *spp. were aligned to create a global phytochrome alignment 358 nucleotides in length, with an average pairwise similarity of 69.4% and 123 identical sites (34.4%). The gene phylogeny generated from this alignment (Figure 6) reflected divergence of *PHYA*, *PHYB*, *PHYC *and *PHYE *as a result of speciation (nodes 1A, 1B, 1C and 1E, respectively) and gene duplication (nodes 2 and 3). The level of nucleotide divergence of each of the gene sub-families after nodes 1A, 1B, 1C and 1E (Kimura 2-parameter distances) was similar, with a mean of 0.297 ± 0.21 nucleotide substitutions per site. However, the synonymous (*K*_*S*_) and non-synonymous (*K*_*A*_) substitution rates were both significantly more variable among the various gene sub-families defined by nodes 1A, 1B, 1C and 1D than were simple nucleotide distances (Table 2). Despite this variation, all sub-families showed a *K*_*A*_*/K*_*S *_ratio <0.1, implying that each remains under purifying selection for function. Further, excessively long branch-lengths, which are often found in pseudogenes, were not observed. In the *PHYB, PHYC *and *PHYE *clades, the branch lengths leading to the Arabidopsis orthologs, which have known biological functions, were longer than the branches leading to their respective cotton orthologs. Considered together, these lines of evidence indicate that each of the phytochrome sub-families retains some biological function in *Gossypium*, as they do in Arabidopsis [14-16,18-31]. Further, our topology supports the conclusion that *PHYD *is the result of a relatively recent gene duplication that may be exclusive to the Brassicaceae family [16].

**Table 2:** Nucleotide divergence in phytochrome genes in comparisons of Arabidopsis and cotton

	K-2P	S Dif	***K***_***s***_	NS Dif	***K***_***A***_	Ka/Ks
**Node 1A**	0.291	46.5	1.82	27.4	0.123	0.068

**Node 1B**	0.296	36.5	1.00	17.5	0.086	0.090

**Node 1C**	0.274	41.0	1.55	30.3	0.147	0.095

**Node 1E**	0.326	49.0	>2.0	23.0	0.122	<0.061

**Node 3**	0.094	17.0	0.309	11.8	0.050	0.163

Nodes refer to the NJ tree in Figure 6. K-2P indicates the mean Kimura 2-parameter distances between Arabidopsis and cotton gene sequences.

## Discussion

### Resolution of the phytochrome gene family

In three out of four cases, we were able to successfully resolve the inventory and evolutionary relationships of the phytochrome genes in diploid and allotetraploid cottons using the hinge region only. This finding supports the general utility of employing the hinge region for identifying GSTs for phytochromes. In only one case (*PHYB*) was additional gene sequence required for sufficient phylogenetic resolution. In another case (*PHYC*), nucleotide divergence at a commonly used primer-binding site prevented the characterization of the hinge region by the typical strategy of using primers based on conserved flanking peptides HYPATDIP and PFPLRYAC. However, nucleotide diversity within the *PHYC *hinge region itself was sufficiently informative to resolve the pattern of evolutionary inheritance through allotetraploidization event.

The sequencing of phytochrome gene fragments from A- and D-genome diploids, as well as from AD allotetraploid taxa, provides an essential foundation for all subsequent analysis of phytochrome function and evolution in *Gossypium*. The sequenced fragments provide sufficient information (at least two diagnostic nucleotide characters) to unequivocally identify or 'tag' various orthologs, homeologs and paralogs, as well as monitor their patterns of nucleotide divergence, and trace their evolutionary inheritance through the allopolyploidization event. This information will serve as a foundation for further sequence assembly and annotation, and will be used to design locus-specific primer sets for quantitative RT-PCR assays that will measure transcript levels for each gene family member. In some cases (e.g. *PHYA1 *vs. *PHYA2*) levels of sequence divergence are high enough to support studies of gene function using RNAi or amiRNA approaches to create gene-specific knockouts [69]. The use of well characterized 'candidate genes' of agronomic interest is becoming an integral component of marker-assisted selection efforts in plants [70]. Several SNP-based molecular markers [71,72] are now being developed using the diagnostic nucleotide characters identified in this study, and are being mapped in experimental cotton populations that show segregation of phytochrome-controlled traits such as fiber length and flowering time.

### The ancestral phytochrome gene complement of the Malvales and Brassicales

Our study indicated that the diploid ancestors to the world's major fiber crops (*G. hirsutum *and *G. barbadense*) had a complement of phytochrome apoprotein genes that was very similar to that of the model plant *Arabidopsis thaliana*. This was not entirely unexpected given the relatively close phylogenetic relationship of the two lineages [64,65]. The most-simple evolutionary scenario is that the last common ancestor of Arabidopsis and cotton, possibly an arborescent species in the late Cretaceous period [65], had a phytochrome gene complement consisting of one functional gene in each of the *PHYA*, *PHYB/D*, *PHYC *and *PHYE *subfamilies.

### *PHYA *duplication in *Gossypium*

After the divergence of the Malvales and Brassicales, the ancestral *PHYA *gene underwent duplication resulting in the observed *PHYA-1 *and *PHYA-2 *paralogs of modern *Gossypium *spp. As the A- and D-genome diploids have both paralogs, the duplication event occurred prior to the divergence of the A- and D-genome lineages. Using 85 MYA (range 68 MYA to 96 MYA) as a rough estimate of the time of divergence of the Malvales and Brassicales [64,73], along with our observed *K*_*s *_of 1.82 in the *PHYA *hinge region in this time interval, we can derive a crude estimate of 0.011 substitutions/synonymous-site/million years, and an estimate of the time of *PHYA *duplication of ~14 MYA. This estimate places the duplication well within the crown group of Malvales and the Malvaceae family [65]. Given our time estimate, the *PHYA *duplication may be exclusive to the genus *Gossypium*, but would have occurred prior to the estimated time of divergence of the A and D genome groups [62]. As neither we nor others [58,62,74] have observed evidence of additional nuclear gene duplications or chromosomal duplications in this time period, the *PHYA *event was likely a tandem or segmental duplication, rather than whole genome duplication.

After a gene duplication event, one of the two newly duplicated genes is theoretically unconstrained by selection for function, and is thus free to accumulate mutations leading to a pseudogene fate, subfunctionalization, or neofunctionalization [75-80]. Although we did not obtain definitive evidence of pseudogenic sequences in any of the phytochromes or taxa studied (e.g. no stop codons or frameshift mutations), we did observe significant variation in *K*_*A*_/*K*_*s *_ratios in pairwise interspecific comparisons (discussed below), leaving open the possibility of pseudogene outcomes. Alternatively, one of the duplicated genes may undergo positive selection to gain a novel function (neofunctionalization). Further, duplicated gene-pairs may subdivide the function of ancestral gene (subfunctionalization). Perhaps the most intriguing fate, which has been observed empirically, but not yet explained in theory, is the situation in which both gene copies may be retained for a lengthy period under what appears to be purifying or negative selection [79,80]. One approach to understanding the evolutionary fates of duplicated genes is through an analysis of the signature of natural selection on amino acid encoding sequences.

Although the hinge regions of phytochromes display relatively high levels of nucleotide diversity [81], they do not evolve under neutrality. The hinge region participates in inter-domain communication in phytochrome molecules [82]. For example, phosphorylation of a serine residue in the *PHYA *hinge plays a likely role in regulating protein-protein interactions between phytochrome and downstream signal-transducing molecules [83]. Compared to wild-type, a mutation in the hinge region of Arabidopsis *PHYB *is deficient in localization into distinct nuclear bodies [84]. Further, a single nucleotide polymorphism (SNP) in the hinge of one of two *PHYB *genes in Aspen (*Populus tremula*, Salicaceae) was associated with natural geographic variation in the timing of bud-set [85].

In comparisons between cotton and Arabidopsis (Table 2), the *K*_*A*_*/K*_*s *_ratio for the *PHYA *hinge region was 0.068 -- a value that is typical for genes under purifying selection [86]. In contrast, the K_A_/K_s _ratio for *PHYA *after gene duplication (node 3) was 0.163, or ~2.4-fold higher. This value is also ~2.1-fold greater than the mean *K*_*A*_*/K*_*s *_ratio of all phytochrome hinge regions (corresponding to nodes 1A, 1B, 1C, and 1D in figure 6) of approximately 0.079 ± 0.014. This significantly elevated *K*_*A*_*/K*_*s *_ratio after the *PHYA *duplication could be attributed to a relaxation of stabilizing selection and/or subfunctionalization of the nascent *PHYA *paralogs (these two alternative possibilities are remarkably difficult to distinguish on the basis of sequence information alone).

The possible functional divergence of *PHYA1 *and *PHYA2 *may be more pronounced after the separation of the A- and D-genome lineages (Table 3). A comparison of *PHYA2 *in the two diploids yields a *K*_*A*_*/K*_*s *_ratio of ~8.2, primarily due to amino acid substitutions in *PHYA2.D*, while *PHYA1 *has a *K*_*A*_*/K*_*s *_ratio of 0.000 in the same taxonomic comparisons. Although this difference is suggestive of possible differential rates of functional evolution in the paralogs, it is not statistically significant in Fisher's exact test (*P = *0.2485). It will be of interest to determine whether the cotton *PHYA *paralogs have distinct functions. Experiments are underway to determine the respective biological functions of each *PHYA-1 *and *PHYA-2 *in *G. hirsutum *and *G. barbadense *using paralog-specific RT-PCR, RNAi gene knockout, and tests for genetic associations between phytochrome-controlled phenotypic traits and *PHYA-1 *and *PHYA-2 *specific molecular markers. A 'candidate gene' approach has recently been used in soy (*Glycine max*) to uncover a genetic linkage between the photoperiod insensitivity locus *E4 *and one of the two the *PHYA *genes, designated *GmphyA1 *and *GmphyA2 *[57]. Loss of photoperiodic flowering is associated with a *Ty1/copia*-like retrotransposon insertion into exon 1 of *GmphyA2*. The authors argue that gene duplication and partial redundancy of the *PHYA *genes may have facilitated the loss of photoperiod sensitivity by allowing the *GmphyA2 (E4) *mutant to avoid the major deleterious phenotypic effects that would have been caused by complete deficiency of *PHYA *gene function.

**Table 3:** Nucleotide divergence in phytochrome genes in comparisons of A- and D-genome derived homeologs in diploid and allotetraploid cottons.

Sequence	Comparison	S dif	S pos	***K***_***s***_	NS dif	NS pos	***K***_***A***_	***K***_***A***_***/K***_***s***_	*P*
PHYA1 Hinge	D-D	2.0	67.33	0.303	0.0	241.67	0.000	**0.000**	**0.049**
	D-T	2.25	67.17	0.102	0.0	241.84	0.000	**0.000**	**0.039**
	T-T	2.5	67	0.038	0.0	242.00	0.000	**0.000**	**0.030**

PHYA2 Hinge	D-D	1.0	66.42	0.015	3.0	242.58	0.125	**8.224**	**0.622**
	D-T	1.0	66.38	0.015	2.0	242.63	0.065	**4.247**	**0.504**
	T-T	1.0	66.33	0.015	1.0	242.67	0.004	**0.270**	**0.386**

PHYB 2.1 kb	D-D	8.0	377.33	0.022	7.0	1293.67	0.005	**0.251**	**0.010**
	D-T	9.0	377.34	0.024	8.0	1293.63	0.006	**0.256**	**0.006**
	T-T	10	377.42	0.027	9.0	1293.58	0.007	**0.300**	**0.004**

PHYC Hinge	D-D	3.0	60.83	0.051	1.0	230.17	0.004	**0.086**	**0.032**
	D-T	3.25	60.67	0.055	1.25	230.09	0.005	**0.103**	**0.035**
	T-T	3.5	60.5	0.06	1.5	230.00	0.007	**0.119**	**0.038**

PHYC 1.0 kb	D-D	7.0	224.67	0.032	3.0	795.33	0.004	**0.120**	**0.002**
	D-T	8.0	224.84	0.037	4.5	795.17	0.006	**0.156**	**0.003**
	T-T	9.0	225	0.041	6.0	795.00	0.008	**0.184**	**0.002**

PHYE Hinge	D-D	4.0	60.42	0.069	1.0	206.58	0.005	**0.071**	**0.012**
	D-T	3.75	60.46	0.065	0.5	206.54	0.003	**0.042**	**0.015**
	T-T	3.5	60.5	0.06	0.0	206.50	0.000	**0.000**	**0.008**

D-D indicates means of comparisons within extant diploids, D-T indicates means of comparisons of extant diploids with tetraploids, T-T indicates means of comparisons within tetraploids. S dif, synonymous differences; S pos, synonymous positions; NS dif, non-synonymous differences; NS pos, non-synonymous positions. *P *indicates significance as determined by Fisher's exact test.

### Persistence and loss of phytochrome paralogs after allopolyploidization

All phytochromes underwent gene duplication by polyploidization at the time of formation of the AD allotetraploids, on the order of 0.5-2.0 MYA [59,61,63,87]. For example, in *G. hirsutum*, we detected a minimum set of ten distinct phytochrome genes, including four *PHYA *genes. In order to assess the evolutionary trajectory of these recently duplicated genes, we examined the synonymous and non-synonymous divergence rates of A- and D-genome phytochrome orthologs and homeologs (Table 3) in pairwise comparisons of 1) diploids with diploids (D-D), 2) diploids with tetraploids (D-T), and 3) tetraploids with tetraploids (T-T). Given that the allotetraploid cottons had both A- and D-genome derived copies of each gene on the order of hundreds of thousands of years, we hypothesized that there may be a relaxation of selection in the allotetraploids, as one of the two copies should no longer be evolutionarily constrained.

However, in comparisons of A- vs. D-genome derived orthologs or homeologs for six GSTs (Table 3), we did not observe dramatic differences in *K*_*A*_/*K*_*s *_between diploid and allotetraploids in any GST except the hinge region of *PHYA2 *(in this case, the observed *K*_*A*_/*K*_*s *_ratio was actually ~30-fold higher in the extant diploids than in the allotetraploids). Because of low levels of nucleotide divergence, we employed Fisher's exact test [88] and found no significant differences in the patterns of nucleotide evolution in allotetraploids vs. diploids. Thus, there was no broad evidence of relaxation of natural selection on gene function after gene duplication by allotetraploidization. Further, the generally low *K*_*A*_/*K*_*s *_ratios across all genes and taxa support a model in which that the phytochrome homeologs are largely evolving independently by a birth-and-death model rather than concerted evolution [89].

The coding sequences of the *PHYB *2.1 kb fragment also appeared be evolving under stabilizing selection in both the diploids (*K*_*A*_/*K_s _*= 0.251) and allotetraploids (*K*_*A*_/*K_s _*= 0.300) reflecting continued selective constraint on coding sequence evolution after polyploidization. However, there was a significant excess of non-synonymous substitutions in both diploids and allotetraploids (*P *= 0.01 and *P *= 0.004, respectively, in Fisher's exact test) indicating a partial relaxation of negative selection and/or functional divergence of the *PHYB *homeologs.

In the allotetraploid cottons, both *PHYC.A *and *PHYC.D *are also evolving in a pattern consistent with purifying selection (*K*_*A*_/*K*_*s *_= 0.184 over 340 codons). However, it should be noted that the *PHYC.D *clade appears to be evolving at distinctly faster rate (8 parsimonious substitutions, including 6 non-synonymous) than the *PHYC.A *clade (2 parsimonious substitutions, both synonymous). This suggests either a relaxation of purifying selection in, or functional divergence of *PHYC.D*. In a similar study of phytochromes in cultivated sorghum (*Sorghum bicolor*) and its wild congeneric relatives [90], *PHYC *was undergoing faster amino acid evolution than *PHYA *or *PHYB*. In the both the *PHYB *and *PHYC *gene subfamilies of cotton, the sequences of the C-terminal signaling domain had higher *K*_*A*_/*K*_*s *_ratios than the corresponding hinge region alone. This may reflect the co-evolution of protein-protein interactions with downstream signaling partners, which are mediated by the C-terminal 'signal transduction' domain [1-6].

While *PHYE-*related contigs had low *K*_*A*_/*K*_*s *_values (0.000 to 0.071), indicating purifying selection, no contig corresponding to an expected *G. barbadense PHYE.A *ortholog was observed. This may have been due to under-sampling of *G. barbadense *clones for sequencing, or due to nucleotide divergence in primer sites (as observed in *PHYC*). Of the 16 *PHYE*-like clone sequences obtained from *G. barbadense*, all were in the D-genome derived clade, which would be an unlikely result (*P *< 0.005, chi-square test) assuming equal amplification efficiencies for *PHYE.A *and *PHYE.D*. Alternatively, the apparent lack of a *PHYE.A *ortholog in *G. barbadense *could be explained by concerted evolution, gene conversion, or by PCR-mediated recombination [66,87]. Overall, the *PHYE *genes, like the other cotton phytochromes, had more synonymous than non-synonymous nucleotide substitutions, favoring a birth-and-death model of gene evolution.

## Conclusions

Our preliminary efforts to obtain an inventory of the cotton phytochrome gene family (based largely on 'hinge' region) indicated that diploid A- and D-genome diploid cottons have two paralogous *PHYA *genes (designated *PHYA1 *and *PHYA2)*, and one each of *PHYB*, *PHYC*, and *PHYE *gene sub-families. Coding sequence evolution in *PHYA2 *was significantly elevated, suggesting loss of selection for function, or incipient subfunctionalization. Other than this duplication and the lack of a separate *PHYD *gene, the phytochrome complement of diploid cottons was very similar to that observed in the closely related model plant *Arabidopsis thaliana*, which greatly facilitates cross-species comparisons.

Whole genome duplication via allopolyploidization (~0.5-2.0 MYA) resulted in additive amalgamation of phytochrome genes within a single nucleus in the allotetraploid, retaining complete gene complements of at least four *PHYA *genes, two genes of each *PHYB*, *PHYC *and *PHYE *in AD-genome *G. hirsutum. G. barbadense *may lack the *PHYE *gene contributed by the A-genome ancestor. Strong purifying selection on nearly all of the phytochrome genes suggests some level of conservation of function of each of the genes after polyploidization. With the possible exception of one of the *PHYE.A *homeologs in *G. barbadense*, we did not see evidence of gene loss. We did not observe any convincing evidence of concerted evolution by gene conversion. Rather, the genes duplicated by allopolyploidy appear to be largely retained, and evolving independently as observed in 48 other nuclear genes in allotetraploid cottons [86].

These results further our understanding of the evolutionary fates of duplicate genes following allopolyploidization. Information on key evolutionary events (such as duplications), as well as rates and patterns of evolutionary change, are an important component of the functional annotation of genes and genomes [91]. These data provide the foundation for more comprehensive studies of the biological functions of each of the cotton paralogs and homeologs. The development of phytochrome 'candidate gene' markers based on the GSTs identified here may prove useful in the mobilization of valuable genes from photoperiodic wild and primitive cottons into elite cotton varieties, in order to improve stress tolerance, disease resistance, fiber quality, and other traits.

## Methods

### Plant Materials

To simplify the assignment of sequences to orthologous or paralogous phytochrome loci (as opposed to alternative alleles at a single locus) we employed diploid and allotetraploid strains that were highly homozygous. Diploid cotton species *G. raimondii *Ulbr. and *G. herbaceum *L. were obtained from the cotton germplasm collection at the Institute of Genetics and Plant Experimental Biology, Tashkent, Uzbekistan. These lines had been maintained by selfing for multiple generations. Genetic standard genotypes *G. hirsutum *L. cv. TM-1 and *G. barbadense *L. cv. 3-79 were obtained from the USDA-ARS Cotton Germplasm Unit, at College Station, Texas, USA. *G. hirsutum *cv. TM-1 [92] is a highly inbred line (>40 generations of selfing). *G. barbadense *cv. 3-79 is a doubled-haploid line [93].

### Genomic DNA isolation and PCR Amplification

Genomic DNAs were isolated from fresh leaf tissue of individual plants from each taxon using the method described by Dellaporta et al. [94]. The primers used in this study (Table 1) were designed using sequences from phytochromes of dicotyledonous plants obtained from the GenBank database http://www.ncbi.nlm.nih.gov and aligned using CLUSTALX software [95]. These included the degenerate primer pair PHYdeg-F/PHYdeg-R, which was designed to amplify the hinge region of the entire phytochrome gene family, and primer pairs PHYABnondeg-F/PHYAdeg-R and PHYABnondeg-F/PHYBdeg-R, designed to amplify the hinge regions of the *PHYA *and *PHYB/D *subfamilies, respectively. In order to amplify additional regions of several the cotton phytochrome genes, degenerate primers that amplify amplicons downstream of the hinge region (in the C-terminal domain) were also designed using this approach. Conserved regions that had approximately 40-55% G+C content were used for primer design. The primer design criteria have been described [96].

PCR reactions were performed in a Robocycler thermocycler (Agilent, USA) with an initial denaturation cycle at 94°C for 3 min., followed by 45 cycles of 94°C for 1 min., 55°C for 1 min. (annealing) and 72°C for 2 min. (extension), followed by a single 5 min. extension at 72°C. A manual 'hot start' cycling protocol was performed through the addition of *Thermus aquaticus *(*Taq*) DNA polymerase in the annealing step of first cycle.

### DNA Sequence analyses

PCR products were cloned into the vector pCR4-TOPO and transformed into *E. coli *TOP10 cells according to manufacturer's instructions (Invitrogen, USA). Cloning was necessary to resolve sequences of duplicated genes. Recombinant plasmids were purified by miniprep (Qiagen, USA) and sequenced using Big-Dye DNA version 1 cycle sequencing chemistry (Applied Biosystems, USA) along with vector-specific forward and reverse primers. As native *Taq *polymerase has an appreciable nucleotide substitution error rate [97], at least 10 clones were sequenced for each amplicon from each diploid taxon, and 20 clones were sequenced from each allotetraploid taxon. Unincorporated dye-labeled terminators were removed from the extension products by Bio-gel P-30 spin column purification (Bio-Rad, USA). Extension products were sequenced using the ABI 310 and ABI3130 Genetic Analyzers (Applied Biosystems, USA).

### Data analyses

Double-stranded, finished sequences for each clone were assembled with Sequencher 4.8 software (Gene Codes, USA). After trimming of vector and amplification primers, sequences were searched against GenBank databases using BLASTN [98]. Searches of the non-redundant nucleotide database (nr) and the *Arabidopsis thaliana *database (Taxid: 3702) were performed using the "discontinuous megablast" method as implemented by the NCBI database [99]. Alignments of clones obtained from each amplicon/taxon combination were performed using ClustalX. Within each taxon, clone sequences were grouped into contigs on the basis of (in all cases) at least two shared diagnostic SNPs and (if present) shared indel polymorphisms. When a single clone differed from other clones in the same consensus contig at a single nucleotide position, these sporadic differences were assumed to be products of *Taq *polymerase substitution error [97].

Consensus sequences were then aligned across all taxa and used for phylogenetic analyses. Distance-based phylogenetic trees were generated using neighbor-joining [100], using a minimum evolution objective, with gaps (indels) ignored, and either uncorrected "p" distances or Kimura two-parameter distances [101], as noted in the figure legends. Parsimony analysis was performed by an exhaustive search implemented by the PAUP software package version 4.0b10 [102]. The robustness of each phylogenetic tree was evaluated by bootstrap replication [103]. Estimates of synonymous substitution rate *K*_*S *_and non-synonymous substitution rate *K*_*A *_were based the Jukes-Cantor correction [104] and calculated by the method of Nei and Gojobori [105] as implemented by the DnaSP ver. 5 software package [106]. The significance of differences in *K*_*A *_and *K*_*S *_were determined by Fisher's exact test [88]. Sequence alignments were scanned for possible recombination using the software package RDP3, employs a suite of recombination detection and analysis methods [67]. Phytochrome ESTs from *Gossypium *spp. were identified in GenBank by searching non-human, non-mouse ESTs (est_others) and *Gossypium *(Taxid: 3633) using the "discontinuous megablast" method as implemented by the NCBI database [99].

## Abbreviations

amiRNA: artificial micro-RNA; bp: base pair(s); FR: far-red light; indel: insertion/deletion polymorphism; *K*_*A*_: non-synonymous nucleotide substitution rate; kb: kilobase(s); kDa: kiloDalton; *K*_*S*_: synonymous nucleotide substitution rate; MYA: million years ago; NJ: neighbor joining; nt: nucleotide; PCR: polymerase chain reaction; R: red light; RNAi: RNA interference; SNP: single nucleotide polymorphism.

## Authors' contributions

IYA and AEP designed the experiment. IYA designed most of the PCR primers and cloned the *PHYA*, *PHYB *and *PHYE *gene families. ZTB performed DNA sequencing of phytochrome genes. CJLY isolated, cloned and sequenced the *PHYC *gene family and participated in the sequencing of *PHYA*, *PHYB *and *PHYE *genes. IYA, AA, CJLY, and AEP performed data interpretation and drafted the manuscript. All authors read and approved the final manuscript.

## Supplementary Material

Additional file 1 **A summary of phytochrome ESTs from Gossypium**. A summary of non-redundant, high-quality ESTs from the GenBank database, accessed on November 15, 2009. HSP: high scoring pair relationship with the corresponding Arabidopsis thaliana ortholog; Min loci: estimate of the minimum number of genomic loci identified by the ESTs (based on sequence differences). Click here for file
