# Supplementary material for: Duplication, divergence and persistence in the Phytochrome photoreceptor gene family of cottons (Gossypium spp.)
Source: BMC Plant Biol. 2010 Jun 20;10:119. doi: 10.1186/1471-2229-10-119 (PMC3095280; doi:10.1186/1471-2229-10-119)
Supplement: Additional file 1 — A summary of phytochrome ESTs from Gossypium. A summary of non-redundant, high-quality ESTs from the GenBank database, accessed on November 15, 2009. HSP: high scoring pair relationship with the corresponding Arabidopsis thaliana ortholog; Min loci: estimate of the minimum number of genomic loci identified by the ESTs (based on sequence differences). [file 1471-2229-10-119-S1.HTML]

HSP


|  |  |  |  |  |
| --- | --- | --- | --- | --- |
| **HSP** | **Taxon** | **ESTs** | **Genbank IDs** | **Min. number loci** |
| PHYA | *G. raimondii* | 4 | CO117336 | 1 |
|  |  |  | CO092073 |  |
|  |  |  | CO092074 |  |
|  |  |  |  |  |
|  | *G. hirsutum* | 5 | ES822585 | 2 |
|  |  |  | EX168627 |  |
|  |  |  | ES823391 |  |
|  |  |  | DV849493 |  |
|  |  |  | DW235365 |  |
| PHYB | *G. raimondii* | 0 | 0 | 0 |
|  |  |  |  |  |
|  | *G. hirsutum* | 3 | DT566665 | 1 |
|  |  |  | ES835169 |  |
|  |  |  | ES850111 |  |
| PHYC | *G. raimondii* | 1 | CO121409 | 1 |
|  |  |  |  |  |
|  | *G. hirsutum* | 0 | 0 | 0 |
| PHYE | *G. raimondii* | 0 | 0 | 1 |
|  |  |  |  |  |
|  | *G. hirsutum* | 2 | DW478704 | 1 |
|  |  |  | DW506498 |  |
